# A Case of an Abdominal Aortic Dissection in a Hemodynamically Stable Marfan Syndrome Patient Presenting without Pain

**DOI:** 10.1155/2020/1704150

**Published:** 2020-02-23

**Authors:** Hussein Al-Mohamad, Kara Stout, Taryn Bolling, Ronald Walsh

**Affiliations:** ^1^Department of Cardiology, Largo Medical Center, Largo, FL, USA; ^2^Department of Internal Medicine, Largo Medical Center, Largo, FL, USA

## Abstract

*Introduction*. Marfan syndrome (MFS) is a rare connective tissue disorder attributed to a defect in the fibrillin-1 gene. Aortic aneurysms and dissection are common causes of morbidity and mortality in Marfan syndrome. *Case Report*. A 43-year-old female with a history of MFS and a 4.0 cm dilated ascending aorta presented to her cardiologist reporting that since a C-section two years prior, the left side of her abdomen painlessly protruded when standing. An outpatient CT scan of the abdomen/pelvis noted a 5.5 cm abdominal aortic dissection, and she was directed to the hospital. Repeat CT scan of the abdomen/pelvis revealed a 5.6 cm dissecting aneurysm of the infrarenal abdominal aorta. The patient was admitted to the ICU and started on a nitroglycerin drip to maintain systolic blood pressure less than 110 mmHg. The patient underwent repair of her abdominal aortic dissection via a retroperitoneal approach, and she tolerated the procedure well. She was started on metoprolol tartrate 12.5 mg BID and aspirin 81 mg postoperatively. She was safely discharged with follow-up care. *Conclusion*. This case stresses the importance of having a low threshold to obtain imaging in a MFS patient with protruding abdomen, even though the patient may not have pain and be hemodynamically stable.

## 1. Introduction

Marfan syndrome (MFS) is a rare connective tissue disorder with an estimated incidence of about 1 in 5,000 persons in the United States; it is generally inherited in an autosomal dominant pattern [1, 2]. The pathogenesis involves a defect in the fibrillin-1 (FBN-1) gene located on chromosome 15 [2–4]. The role of FBN-1 is to provide structural support to tissues and to regulate elastogenesis [5]. Defects in this gene can lead to mitral valve prolapse (MVP), joint laxity, and ectopia lentis [5]. More serious clinical findings include aortic root dilatation and thoracoabdominal aortic aneurysms and dissections [2]. Annual echocardiograms are recommended for patients with known MFS to monitor for aortic root dilatation [6].

Several cases have been reported about abdominal aortic aneurysms, dissections, and ruptures [2, 3, 7–9]. In review of the literature, it is rare to find a case of a hemodynamically stable and fairly asymptomatic abdominal aortic dissection in a patient with MFS. We present such a case to increase awareness about this rare, yet severe complication.

## 2. Case Report

We present a case of a 43-year-old nonsmoking female with a past medical history of MFS, MVP, and a 4.0 cm dilated ascending aorta who presented to her cardiologist after being diagnosed with an abdominal aortic dissection by her OB-GYN. The patient noted that since a C-section 2 years prior, the left side of her abdomen protruded more when standing compared to the right. She denied associated abdominal, chest, or back pain. The patient was told that she may have a hernia in the past.

Because she was concerned about the asymmetry of her abdomen, she went to a new OB-GYN who ordered an abdominal CT scan of the abdomen/pelvis with IV contrast which showed a 5.5 cm abdominal aortic dissection. The patient was reluctant to go to the hospital until she saw her cardiologist a few days later, who recommended hospitalization and a consult for cardiothoracic surgery.

**Figure 1:**
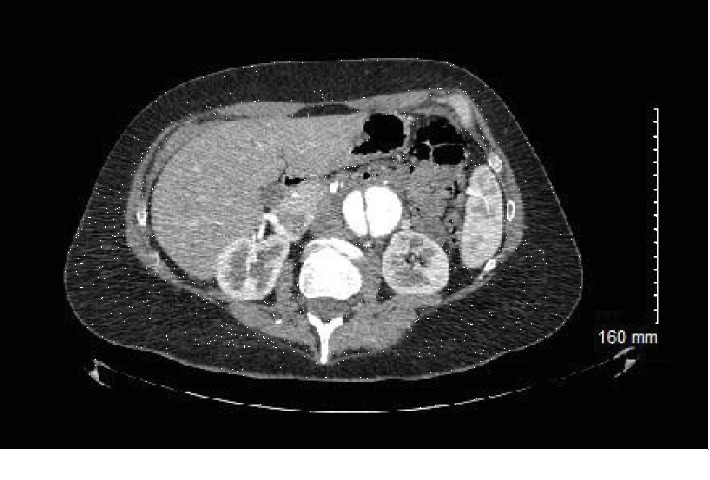
CT scan of the abdomen/pelvis with IV contrast demonstrating dissection of the abdominal aorta.

Interestingly, when the patient was seen in the hospital, she again denied chest pain, back pain, abdominal pain, dyspnea, nausea, vomiting, and dizziness. She only mentioned that her left side of the abdomen protrudes more than the right side when standing. On physical exam, patient had several features typical to MFS: she was tall, had a high arched palate, arachnodactyly, pectus excavatum, and her arm span was greater than her height. Her abdomen was soft, nontender, nondistended, had normal bowel sounds, and no pulsatile mass was appreciated. However, when the patient stood up, it was clear that the left side of her abdomen was bulging out in comparison to the right. A repeat CT scan of the abdomen/pelvis revealed a dissecting aneurysm of the infrarenal abdominal aorta measuring up to 5.6 cm (Figure 1); the false lumen supplied the left common iliac artery and the true lumen supplied the right common iliac artery. There was no evidence of thoracic aortic dissection.

The patient was admitted to the ICU and started on a nitroglycerin drip to maintain a systolic blood pressure less than 110 mmHg. Cardiothoracic surgery was consulted and recommended the patient to undergo open surgical repair of the abdominal aortic dissection. In order to risk stratify the patient prior to the procedure, the patient underwent a regadenoson nuclear medicine stress test, which was low risk for ischemia. The patient underwent successful repair of her abdominal aortic dissection via a minimally invasive open retroperitoneal approach. She tolerated the procedure well without postoperative complications.

The patient came into the hospital with no home medications. As she has reported in the past, she was tried on nebivolol but had failed it due to syncope. Given this, metoprolol tartrate 12.5 mg PO BID was started cautiously to provide long-term benefits for the patient's abdominal and thoracic aorta. The patient tolerated this medication well and was safely discharged home with close cardiovascular follow-up.

## 3. Discussion

Aortic root disease leading to aneurysmal dilation, regurgitation, and dissection is the most common cause of morbidity and mortality in MFS [10]. Aortic aneurysms and dissections arise due to the fragility of the aortic tissues, which are responsible for the rise of cystic medial necrosis [11].

Class I screening guidelines by the American College of Cardiology (ACC) include an echocardiogram at the time of diagnosis of MFS to determine the diameter of the aortic root and ascending aorta. Repeat imaging is recommended at six months and then annually to follow the rate of enlargement. If the aorta is greater than 4.5 cm or has significantly changed from baseline, more frequent screening is recommended [12].

Pregnancy in women with MFS can be challenging and is associated with an increased risk of aortic dissection. Recommendations include preconception counseling, TTE, and initiation of a protective beta blocker to slow the growth of the aortic root [13]. Serial TTE's are performed every 4-12 weeks to monitor aortic size, and C-section is recommended for aortic root dilation over 4 cm [13]. For aortic root dilatations over 4.5 cm, prophylactic aortic root and ascending aorta replacement is recommended prior to pregnancy [13]; but, even after successful root replacement, these patients remain at high risk for future events [14]. In our case, the patient followed the pregnancy guidelines and delivered her child via a C-section.

While MFS commonly leads to aortic root aneurysm and descending thoracic aortic aneurysms, the presence of an abdominal aortic aneurysm (AAA) and abdominal aortic dissection is rarely reported [3, 4, 7–9]. Up to 20% of patients with infrarenal AAAs have a family history of AAAs, suggesting there is a likely inherited component. Routine spiral thin-slice CT angiography or magnetic resonance angiography imaging of the entire aorta is recommended if there is enlargement of the descending thoracic aorta and abdominal aorta or if there is aortic dissection [6, 15].

Strategies to combat the increased risk of dissection include life-long *β*-blockade and elective aortic root replacement at 5 cm [2]. In addition, low-dose aspirin therapy helps to reduce major coronary events, cardiovascular-related mortality, and ischemic stroke risk [16]. Blood pressure management with a beta blocker up titrated to a goal heart rate less than 100 beats per minute with exercise is initiated to reduce cardiovascular mortality [15]. In our case, the patient was started on metoprolol tartrate and low-dose aspirin to maximize risk reduction for future events.

A decision to repair an AAA is also not MFS specific. It is based on size greater than or equal to 5.5 cm in males, 5 cm in females, or expansion of more than 5 mm in 6months or 1 cm over one year [16]. Risk factors for rupture include advanced age, male sex, Caucasians, smoking, other large vessel aneurysms, and atherosclerosis [16, 17].

The decision to repair a dissection depends on the type of dissection but is also not specific to MFS. Stanford Type A dissections are a surgical emergency [18]. For Stanford Type B dissections, medical treatments are initially attempted with esmolol or labetalol to keep systolic blood pressure between 100 and 120 mmHg and nitroprusside for vasodilation [18]. In Type B dissections, indications for surgery include the evidence of end-organ malperfusion, refractory pain, a rapidly expanding false lumen, impending or frank rupture, or a chronic aneurysmal dilation with a diameter of greater than or equal to 5 cm [18]. In our case, the patient met the final criteria of chronic aneurysmal dilation over 5 cm. Nitroprusside was used for both blood pressure management and vasodilation. Perioperative management includes an assessment for underlying coronary artery disease and ischemia if needed [6]. In this case, our patient underwent a regadenoson nuclear medicine stress test prior to her surgical procedure.

In general, endovascular techniques were not initially intended for thoracic and abdominal aorta pathology in patients with connective tissue diseases [14, 19]. Patients specifically with MFS were not included in initial endograft studies due to the fragile nature of the marfanoid aorta and concern for the predictive ability of the long-term radial force of the endograph to provide a seal without adversely damaging the vessel [14, 19]. Furthermore, as patients are typically young and with unclear long-term durability of current stent grafts, these should be avoided [14]. The European Society of Vascular Surgery guidelines recommend an open surgical procedure, with considerations for EVAR only if the patient is deemed too high of a risk for the open intervention [14] or if EVAR is a life-saving bridging procedure until definitive open repair can be safely performed [19]. In accordance, a minimally invasive retroperitoneal approach for open AAA repair was performed for our patient, and she tolerated the procedure without major complications.

## 4. Conclusion

In conclusion, this case stresses the importance of having a low threshold to obtain imaging in a MFS patient with bulging or protruding abdomen, even though, the patient may not have pain and be hemodynamically stable.
